# Supplementary material for: Variabilities in N2 and E Gene Concentrations in a SARS-CoV-2 Wastewater Multiplex Assay
Source: Microorganisms. 2025 Aug 9;13(8):1862. doi: 10.3390/microorganisms13081862 (PMC12388642; doi:10.3390/microorganisms13081862)
Supplement: Supplementary file 1 [file microorganisms-13-01862-s001.zip › microorganisms-3765701-supplementary.pdf]

# Supplementary Materials

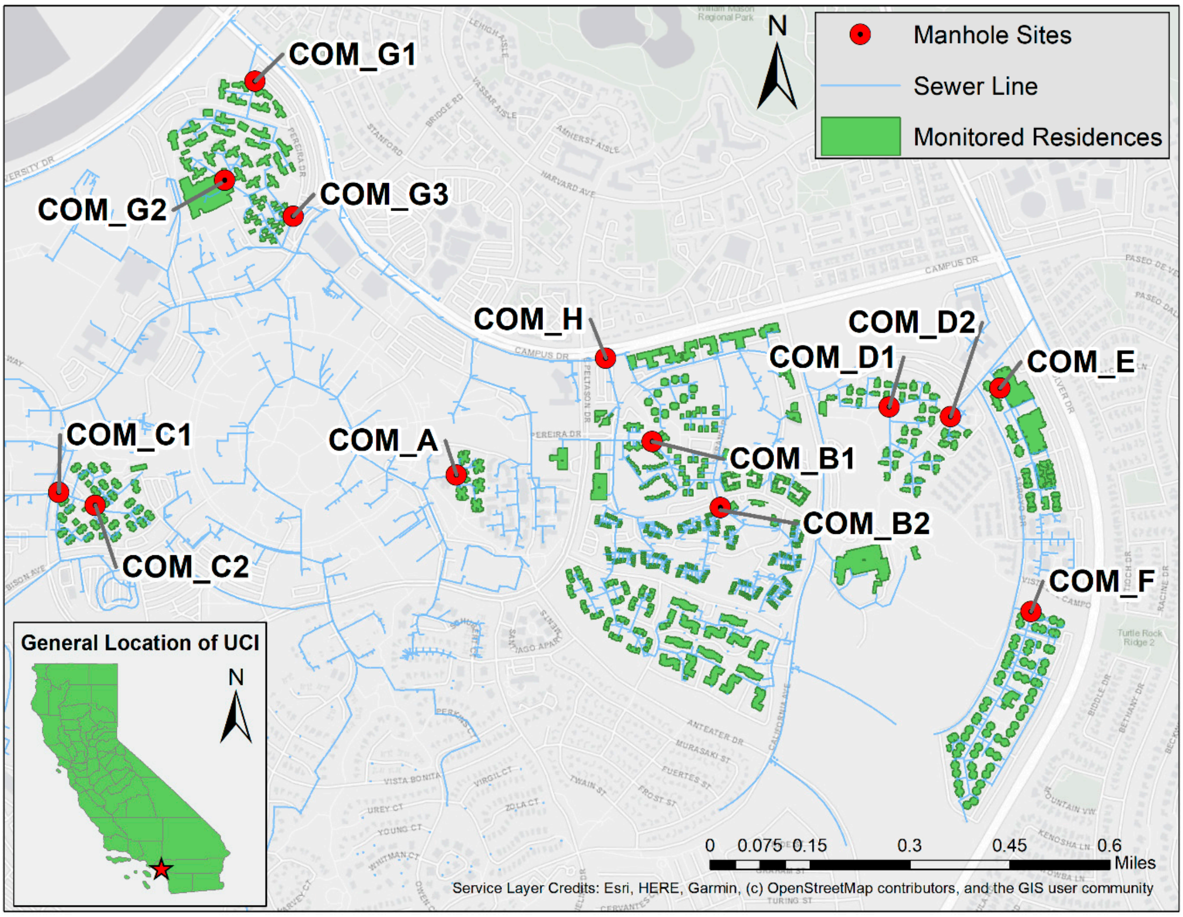


**Figure S1.** Campus map depicting where the COM_H site is located to receive significant wastewater flows from a large portion of the UCI campus.

**Table S1.** Multiple regression results from testing MHV recovery, TSS, and COD effect on variance of the SARS-CoV-2 N2 and E gene concentrations.

| **N2 GENE** | | | | | |
|---|---|---|---|---|---|
| Predictor | Estimate | Std. Error | t value | p-value | Explanation |
| MHV Recovery Efficiency | -1185 | 532.2 | -2.227 | 0.0263 | Significant negative effect on N2 |
| TSS (mg/L) | -0.02225 | 0.2559 | -0.087 | 0.931 | Not significant |
| COD (mg/L) | 0.1656 | 0.16 | 1.035 | 0.301 | Not significant |
| **E GENE** | | | | | |
| MHV Recovery Efficiency | -417.48 | 516.98 | -0.808 | 0.4197 | Not significant |
| TSS (mg/L) | 0.2576 | 0.2486 | 1.036 | 0.3004 | Not significant |
| COD (mg/L) | 0.195 | 0.1554 | 1.255 | 0.21 | Not significant |

Multiple $R^2$ = 0.19 for N2, 0.27 for E

**Table S2.** Multiple regression results from testing MHV recovery, TSS, and COD effect on variance in the SARS-CoV-2 N2 and E gene concentrations. For the second test, “low water quality” and “high water quality” groups were designated.

| | | | | | | | |
|---|---|---|---|---|---|---|---|
| Low Water Quality Model* | N2 | | | | | | |
| | | **Predictor** | **Estimate** | **Std. Error** | **t value** | **p-value** | **Explanation** |
| | | MHV Recovery Efficiency | -1019.5 | 883.83 | -1.15 | 0.25 | Not significant |
| | | TSS (mg/L) | | 1.97 | 1.68 | 0.095 | Not significant |
| | | COD (mg/L) | | 0.91 | -1.31 | 0.194 | Not significant |
| | | **Multiple $R^2$** | **0.006** | | | | |
| | E | | | | | | |
| | | MHV Recovery Efficiency | -279.44 | 1044.86 | -0.27 | 0.789 | Not significant |
| | | TSS (mg/L) | 4.4 | 2.32 | 1.89 | 0.06 | Not significant |
| | | COD (mg/L) | -0.52 | 1.08 | -0.48 | 0.633 | Not significant |
| | | **Multiple $R^2$** | **0.009** | | | | |
| High Water Quality Model** | N2 | | | | | | |
| | | MHV Recovery Efficiency | -6074 | 3813 | -1.59 | 0.114 | Not significant |
| | | TSS (mg/L) | -0.09 | 0.44 | -0.2 | 0.845 | Not significant |
| | | COD (mg/L) | -0.01 | 0.29 | -0.03 | 0.977 | Not significant |
| | | **Multiple $R^2$** | **0.001** | | | | |
| | E | | | | | | |
| | | MHV Recovery Efficiency | -5672 | 3177 | -1.79 | 0.077 | Not significant |

| | | | | | | | |
|---|---|---|---|---|---|---|---|
| | | TSS (mg/L) | 0.31 | 0.37 | 0.84 | 0.401 | Not significant |
| | | COD (mg/L) | -0.03 | 0.24 | -0.14 | 0.886 | Not significant |
| | | **Multiple $R^2$** | **0.031** | | | | |

*Low water quality: TSS>300mg/L, COD>1000mg/L, MHV<25%

**High water quality: TSS<300mg/L, COD<1000mg/L, MHV>25%

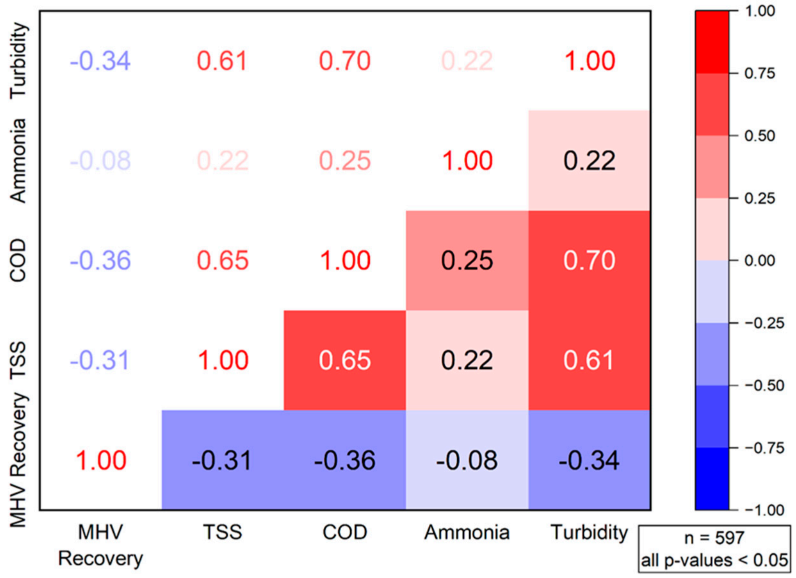


**Figure S2.** Kendall's Tau Correlation Coefficient Matrix for water quality parameters and MHV.

**In-house modified SOP for ASTM D5907 Standard Methods for Non-Filterable Matter.**

**TSS Instructions for Wastewater Samples**
**Preparation of Filters**

1. Obtain 24 filters (1.5 um pore size, 47 mm diameter glass fiber) and lay out on aluminum weighing dishes with wrinkled sides up.
2. Run 150 mL of DI/Milli-Q water through the filter using a vacuum filtration apparatus.
3. Leave the vacuum on for at least 20 seconds to remove any excess moisture. Tilt filter holder gently to obtain any water off the sides of the apparatus.
4. Take filters to room 837 for baking in a muffle furnace at 105°C for around 1 hour.
5. Remove aluminum dish with filters from oven carefully and transfer to desiccator for at least 30 min. Can leave for multiple days until filters need to be used.

**TSS procedure (2 blanks + 11 samples in replicate)**

1. Obtain 24 filters (1.5 um pore size, 47 mm diameter glass fiber) and lay out on aluminum weighing dishes with wrinkled sides up.
2. Weigh dish + filter on analytical scale and record weights.

*Blank procedure (2 blanks)*

3. Run 150 mL of DI/Milli-Q water through the filter using a vacuum filtration apparatus.
4. Leave the vacuum on for at least 20 seconds to remove any excess moisture. Tilt filter holder gently to obtain any water off the sides of the apparatus.
5. Remove the filter and repeat for a second blank.

*Wastewater sample procedure*

6. Run 50 mL of DI/Milli-Q water through a filter using a vacuum filtration apparatus.
7. Obtain a defrosted wastewater sample and mix vigorously to homogenize the sample. Pour half of the wastewater sample (usually around 20-25 mL) onto the filter paper slowly and allow it to drain through the filter. Record how much volume of sample was filtered in the lab notebook. Reserve the other half of the wastewater sample for a second replicate. All samples are to be performed in replicate.
   *Note: if there is not a full sample available for TSS testing, use whatever volume is available, ensuring at least 10 mL of sample is used for each replicate.*
8. Run another 50 mL of DI/Milli-Q water through the filter to thoroughly saturate the filter and ensure all solids are on the filter and not the filter holder.
9. Leave the vacuum on for at least 20 seconds to remove any excess moisture. Tilt filter holder gently to obtain any sample off the sides of the apparatus.
10. Transfer the filter to the aluminum dish carefully. Repeat for replicate with remaining sample and then repeat for all other filters.
11. Take filters to room 837 for baking in a muffle furnace at 105°C for around 1 hour.
12. Remove aluminum dish with filters from oven carefully and transfer to desiccator for at least 30 min.

13. Weigh dish + filter on analytical scale after baking and desiccating, and record weights.
14. Add measurements to the master data sheet and calculate TSS.
